# Integrated data-independent acquisition and thermal proteome profiling for proteomic characterization of lamotrigine-treated MCF-7 cells

**DOI:** 10.1007/s00216-026-06540-z

**Published:** 2026-05-20

**Authors:** Annarita Giuliano, Elena Ricci, Caterina Gabriele, Mariarosa Fava, Sofia Spadafora, Catia Morelli, Diego Sisci, Marco Gaspari

**Affiliations:** 1 Research Centre for Advanced Biochemistry and Molecular Biology, Department of Experimental and Clinical Medicine, Magna Græcia University of Catanzaro, 88100 Catanzaro, Italy; 2 Department of Pharmacy, Health and Nutritional Sciences, University of Calabria, 87036 Rende, Italy

**Keywords:** Lamotrigine, Breast cancer, Drug repurposing, Proteomics, Data-independent acquisition (DIA), Thermal proteome profiling (TPP)

## Abstract

Lamotrigine, a clinically approved anticonvulsant, has recently gained interest for its potential anticancer effects. However, the molecular mechanisms underlying its activity in breast cancer remain poorly understood. Here, an integrated mass spectrometry–based proteomic strategy combining data-independent acquisition (DIA) and thermal proteome profiling (TPP) was applied to investigate the cellular effects and potential targets of lamotrigine in the human breast cancer cell line MCF-7. The DIA-based quantitative approach identified 6622 proteins, of which 142 were significantly modulated following lamotrigine exposure. Differentially expressed proteins were mainly associated with mitochondrial energy metabolism, chromatin organization, and protein translation. TPP experiments identified significant thermal stability alterations in ABRAXAS2, MT-CYB, TMEM97, and MTA2 upon lamotrigine treatment. Notably, both DIA and TPP analyses highlighted mitochondrial alterations, with consistent involvement of MT-CYB, suggesting mitochondrial dysfunction as a potential central mechanism of lamotrigine’s action. Overall, these findings characterize proteome-level alterations induced by lamotrigine and support its further evaluation in breast cancer.

**Graphical abstract:**

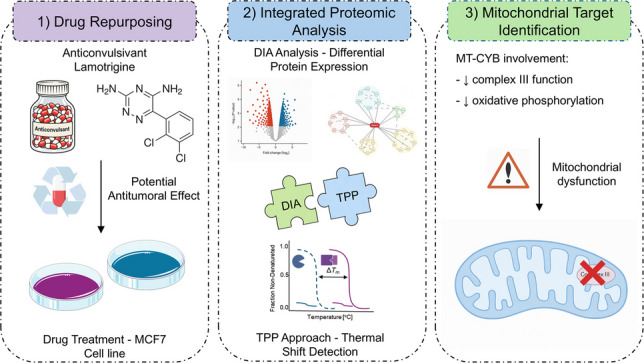

**Supplementary Information:**

The online version contains supplementary material available at 10.1007/s00216-026-06540-z.

## Introduction

Breast cancer remains a major global health challenge, accounting for 30% of all new cancer diagnoses and 15% of cancer-related deaths among women [1, 2]. Despite significant progress in early detection and treatment, the complex pathogenesis and clinical heterogeneity of breast cancer continue to hinder effective prevention and therapeutic success [3].

Current treatment strategies are primarily based on the tumor’s histopathological and molecular characteristics, with standard approaches including surgery, chemotherapy, radiotherapy, endocrine therapy, and targeted agents [4]. Although these interventions have improved patient outcomes, their clinical effectiveness is often compromised by toxicity, adverse effects, and the development of multidrug resistance, particularly to antioestrogen therapies [5]. These limitations underscore the need to explore alternative therapeutic options with improved efficacy and a lower tendency to induce resistance [6].

In response to these challenges, significant efforts have been directed toward developing novel anticancer agents, leading to the identification of numerous compounds that target distinct molecular pathways and exhibiting a range of preclinical and clinical efficacy [7]. However, drug discovery is a complex, multi-stage process that includes target identification and validation, lead compound screening and optimization, preclinical studies, and ultimately, clinical trials to evaluate safety and efficacy in humans [8]. Up to 15 years and substantial financial investment are required to advance a drug from the laboratory to clinical application. Despite this, fewer than 10% of candidate drugs reach the market [9].

To address this concern, drug repurposing has gained growing interest as a promising alternative. By identifying new therapeutic applications for already approved compounds, this approach can significantly reduce both development time and costs, while benefiting from existing safety and pharmacological data to accelerate clinical translation [10, 11]. In this context, antiepileptic drugs (AEDs) have emerged as particularly attractive candidates among the various pharmacological classes considered for repurposing in oncology [12]. Initially developed to manage epilepsy and other neurological conditions, several AEDs have shown potential antitumor activity through mechanisms involving apoptosis induction, inhibition of cell proliferation, modulation of oxidative stress, and epigenetic regulation [13]. Notably, compounds such as valproic acid, carbamazepine, phenytoin, and lamotrigine have been investigated for their ability to interfere with tumor progression through distinct mechanisms of action [14].

Among these compounds, lamotrigine has attracted considerable attention for its potential application in breast cancer therapy. Its primary mechanism of action involves the inhibition of voltage-sensitive sodium channels, leading to stabilization of presynaptic neuronal membranes and reduced glutamate release [15]. In addition, lamotrigine has been shown to block voltage-dependent calcium channels, particularly N- and P/Q/R-type, while exerting minimal or no activity on L-type channels [16]. Beyond its established neurological effects, recent preclinical studies have highlighted lamotrigine’s ability to inhibit cell proliferation and restore endocrine sensitivity in hormone-resistant breast cancer cells, including tamoxifen-resistant MCF-7 models [17]. Moreover, emerging evidence suggests a role in histone deacetylase (HDAC) inhibition, epigenetic modulation, and suppression of the PI3K/AKT signaling pathway [16].

Despite encouraging preclinical findings, lamotrigine has not yet been included in clinical breast cancer treatment, either as monotherapy or in combination with existing therapies. Further investigations are required to define the antitumor potential of lamotrigine and to obtain a comprehensive molecular signature of the associated cellular response.

Since pharmacological effects are often reflected by changes in protein expression and function, proteomic analysis represents a powerful tool to investigate the molecular alterations induced by lamotrigine in breast cancer models [18]. In this context, advances in mass spectrometry–based proteomics have enabled the comprehensive identification and quantification of proteins in complex biological systems, offering valuable insights into drug-induced cellular responses, signaling pathways, and potential therapeutic targets [19, 20].

Among quantitative proteomic strategies, data-independent acquisition (DIA) has gained wide implementation due to its high reproducibility, broad dynamic range, and ability to consistently quantify thousands of proteins in complex biological matrices [21, 22].

In parallel, the application of chemical proteomics approaches in drug research has expanded rapidly, driven by the need to characterize direct drug-protein interactions and phenotypic effects beyond changes in protein abundance [23, 24]. Among these, thermal proteome profiling (TPP) has emerged as a powerful technique for assessing alterations in protein thermal stability, providing insights into target engagement and mechanisms of action in a cellular context [25, 26].

In the present study, a combination of DIA strategy and TPP approach was applied to characterize the proteomic alteration induced by lamotrigine in the human breast cancer cell line MCF-7, aiming to elucidate its molecular targets and downstream effects.

## Experimental procedures

### Reagents

All chemicals were purchased from Sigma-Aldrich (St. Louis, MO) unless otherwise specified.

### DIA-based proteomic profiling

#### Cell culture and protein extraction

ER+ human breast cancer epithelial cell line (MCF-7) was grown in DMEM/F-12 medium, supplemented with 5% fetal bovine serum (FBS), 100 IU/mL penicillin, 100 ng/mL streptomycin, and 0.2 mM l-glutamine. Cells were seeded and treated with 50 μM of lamotrigine for 24 h and subsequently lysed using the following cytosolic lysis buffer: 50 mM HEPES (pH 7.5), 150 mM NaCl, 1% Triton X-100, 1.5 mM MgCl₂, 10 mM EGTA (pH 7.5), 10% glycerol, and protease inhibitors (0.1 mM sodium orthovanadate, 1% PMSF, and 20 µg/mL aprotinin), supplemented with 1% NP-40.

#### Sample preparation for MS analysis

Protein concentration was estimated using the Qubit Protein Assay Kit (Thermo Fisher Scientific), following the manufacturer’s instructions.

Forty micrograms of protein extract was diluted with lysis buffer to achieve an equal starting protein concentration (2 μg/μL) for both conditions. An aliquot of 20 µL of lysate was brought to a final volume of 50 µL using a denaturing buffer consisting of 0.85% SDS and 170 mM Tris-HCl (pH 8).

Reduction and alkylation of disulfide bonds were performed by adding five microliters of 100 mM dithiothreitol (DTT) and six microliters of 200 mM iodoacetamide (IAA), respectively; each step involved 1 h of incubation on a thermomixer at 37 °C under gentle agitation (650 rpm). To quench residual iodoacetamide, one microliter of 100 mM DTT was added and the reaction was allowed to proceed for thirty minutes at 37 °C.

Protein digestion was carried out according to the protein aggregation capture (PAC) protocol [27]. For each condition tested, two technical replicates were processed as follows: one hundred μL of MagReSyn® hydroxyl microparticles was transferred to a fresh 2-mL tube and was placed on a magnetic rack. Beads were retained by a magnet, and the storage solution was discarded. Beads were equilibrated by adding one hundred μL of 70% acetonitrile (ACN). The suspension was mixed by gentle agitation to avoid particles from settling. Wash solution was removed after placing the tube on the magnet. This procedure was repeated once for a total of two equilibrations. The equilibrated beads were reconstituted in equilibration solution to obtain a concentration of twenty μg/μL. To each reduced and alkylated protein mixture (20 μL), a 5-μL aliquot of beads was added and protein aggregation was induced by adding pure acetonitrile to reach a final concentration of 70% (v/v). To ensure a uniform bead suspension during protein aggregation and precipitation, samples were incubated at room temperature for ten minutes with mixing at 1100 rpm in a thermomixer. Following binding, tubes were held to the magnetic rack, and the supernatant was removed. Proteins bounded on beads were washed three times with ACN followed by one wash with 70% ethanol. Wash procedure was performed on the magnetic rack.

For digestion, samples were removed from the magnetic rack and rinsed beads were reconstituted in fifty μL of 50 mM ammonium bicarbonate (TEAB) pH 8.5 aqueous buffer. Two hundred nanograms of trypsin was added to each sample and allowed to react overnight at 37 °C on a shaker operating at 1100 rpm.

The supernatant containing the digested peptides was harvested and beads were incubated again with fifty microliters of 0.1% FA (2 min at RT, 1100 rpm) to collect any residual peptides. The two supernatants were pooled together.

#### LC-MS/MS analysis

Peptides were separated using a Vanquish Neo UHPLC system (Thermo Fisher Scientific) coupled to an Orbitrap Exploris 240 mass spectrometer (Thermo Fisher Scientific) equipped with a nano-spray ESI source operating at positive polarity at 1.90 kV.

Peptides were loaded directly onto a Thermo Scientific™ PepMap™ Neo analytical column (75 μm × 15 cm, 2 μm particle size) connected to a Thermo nanoViper™ emitter.

The gradient was generated using mobile phase A (0.1% FA, 2% ACN) and mobile phase B (0.1% FA and 80% ACN). Mobile phase B went from 5 to 20% in 38 min, from 20 to 35% in 19 min, from 35 to 55% in 1 min, and from 55 to 100% in 2 min. The column was cleaned for 5 min with 100% of B. The DIA method consisted of a MS1 scan of 370–900 m/z at a resolution of 60,000, an AGC target of 100, and a maximum injection time of 50 ms, followed by 20 sequential MS2 windows acquired at 60,000 resolution, with an AGC target of 1000 and a maximum injection time of 120 ms. In detail, the 20 windows enclosed 4 windows with an isolation window of 30 m/z, 13 windows with an isolation of 20 m/z, and 3 windows with an isolation window of 50 m/z; the overlap for each window was equal to 1 m/z. This acquisition scheme resulted, after database searching, in the identification of a median of 3687 precursors per window (IQR, 3010–4271; range, 2523–5679).

#### Data processing

LC-MS/MS raw data were processed using Spectronaut (Biognosys, version 19) in DirectDIA mode. Search parameters included a maximum of two variable modifications per peptide, with carbamidomethylation set as a fixed modification and N-terminal acetylation and methionine oxidation as variable modifications. Peptide-level missing values were imputed using the Global Imputation algorithm.

Protein identification was performed against a *Homo sapiens* reference database containing one protein sequence per gene, downloaded from UniProt on March 18, 2022 (20,577 entries).

Data processing and visualization were performed in R (version 4.4.2) using the RStudio environment (version 2024.12.0.467). Differential protein expression analysis was carried out with the MS-DAP R package [28].

Data exported from Spectronaut, including raw peak area values used for normalization and statistical modeling, were imported into the MS-DAP workflow. Technical replicates corresponding to the same biological sample were merged, and only features consistently quantified in both replicates were retained for downstream analysis to enhance data robustness.

MS-DAP supports the use of multiple statistical models for differential expression analysis (DEA) [29]. In this study, three models were applied: DEqMS, MS-EmpiRe, and MSqRob. A protein was considered differentially expressed if identified as significant by at least two out of the three models.

All figures were generated using the ggplot2 package (version 3.2.2), embedded within the tidyverse framework (version 2.0.0), in R (version 4.4.2).

Heatmaps of differentially expressed proteins were created using the pheatmap package (version 1.0.13). Gene Ontology (GO), Kyoto Encyclopedia of Genes and Genomes (KEGG), and Reactome pathway enrichment analyses were performed using the clusterProfiler R package (version 4.14.6). Differentially expressed proteins were functionally annotated to identify significantly enriched biological processes, molecular functions, cellular components, and biological pathways.

Protein-protein interaction (PPI) analysis was performed using the STRING database (version 12; https://string-db.org). The list of differentially expressed proteins was submitted to STRING, and interactions were retrieved using a confidence score threshold of 0.4, considering only interactions based on experimental evidence and curated databases. The resulting interaction network was exported and visualized in Cytoscape (version 3.10.3).

### Thermal proteome profiling analysis

#### Thermal shift assay

MCF-7 cells were cultured as previously described, and intact-cell TPP experiments were carried out according to previously established procedures [30]. Briefly, MCF-7 cells were treated with fifty μM of lamotrigine or an equivalent amount of vehicle in complete media for thirty min or twenty-four h at 37 °C in a humidified 5% CO_2_ atmosphere. Following the incubation, cells were collected at 1800 rpm and 4 °C for two min and washed with ice-cold PBS. Washed cells were resuspended in 1.2 mL of PBS and then 100-µL aliquots of the suspension were transferred into 0.2-mL PCR tubes for thermal profiling. Each fraction was heated in parallel in a PCR block for three min at the reported temperature (37, 41, 44, 47, 50, 53, 56, 59, 63, and 67 °C) followed by incubation for three min at room temperature. Thirty µL of ice-cold PBS supplemented with 1% NP-40 and protease inhibitors was added to the samples. Cells were lysed by performing two freeze-thaw cycles according to a previously reported procedure. In detail, cells were snap-frozen in liquid nitrogen for one min, briefly thawed at 25 °C, and transferred onto ice. The entire content was then centrifuged at 13200 rpm for twenty min at 4 °C to separate the soluble protein fraction from the aggregated proteins. After centrifugation, thirty µL of the supernatant was transferred into a new tube and stored at −80 °C until further prepared for MS analysis.

#### Sample preparation for mass spectrometry

##### Reduction and alkylation of protein extracts

Protein extract denaturation and reduction were performed by adding to each sample 7.5 μL of the following buffer: 1% sodium dodecyl sulfate (SDS), 50 mM dithiothreitol (DTT), and 500 mM Tris buffer at pH 8.5. Samples were heated for twenty minutes at 56 °C under gentle agitation (650 rpm on a thermomixer). To prevent disulfide bond reformation, nine μL of 100 mM iodoacetamide (IAA) was added, and the mixtures were incubated for one hour at 37 °C in the dark. Subsequently, the reaction was quenched through the addition of 1.5 μL 50 mM DTT, and the incubation was allowed to proceed for twenty minutes at 37 °C.

##### Estimation of protein quantity by NanoDrop™

The protein concentration of samples treated at 37 °C and 41 °C was measured at 280 nm by a NanoDrop™ 1000 spectrophotometer (Thermo Fisher Scientific, Inc., Waltham, MA, USA). For this purpose, 1 μL of the reduced and alkylated samples was digested according to the PAC protocol as previously described, with some modifications. The protein digestion was performed in 25 μL ammonium bicarbonate (TEAB) pH 8.5 aqueous buffer and 200 ng of trypsin was added. Samples were incubated for 1 h at 47 °C in a thermomixer at 1100 rpm. Subsequently, the supernatant was harvested and evaporated at 30 °C in a speed-vac system. Peptide mixtures were resuspended in 5 μL of a solution consisting of 2% acetonitrile (ACN) and 0.1% formic acid (FA).

To estimate the protein amount, a calibration line was constructed by preparing seven different solutions (25, 50, 75, 100, 150, 200, and 400 ng/µL) of a HeLa digest stock (400 ng/µL, Thermo Fisher Scientific). To ensure proper drop formation on the pedestal, a volume equal to 2 μL was used and the measurement was performed in duplicate. The average measured concentration values were then plotted against the respective theoretical protein quantities. The resulting calibration curve was then used to estimate protein concentrations from the samples treated at 37 °C and 41 °C. The average of the two samples was taken as the initial protein concentration. A volume containing 10 μg of proteins based on the initial protein concentration was then withdrawn from each of the 10 aliquots subjected to increasing temperature and transferred to a new tube. Protein digestion was subsequently performed as follows.

##### Protein digestion and TMT labeling

Protein digestion for TPP experiments was also performed using the protein aggregation capture (PAC) protocol, following the same procedure described for the DIA experiments.

Following digestion, TMT labeling was carried out by adding twenty microliters of TMT reagent directly to the peptide mixtures. Samples were incubated for 60 min (RT, 800 rpm) and the reaction was quenched by using a final concentration of 0.1% hydroxylamine. Finally, beads were separated using a magnetic rack and the labelled peptide solution was collected.

Labelled peptides of the same batch were pooled into a single tube. Multiplexed samples were dried and resuspended in 200 μL of 0.1% TFA. To reduce the complexity of the peptide mixture and increase the number of protein identifications, tip-based high-pH reversed-phase fractionation was performed. StageTips were placed into 200-μL pipette tips, and the equilibration and wash steps were carried out as previously described [31]. Three different StageTips were used for each sample. The peptides were stepwise eluted from the resin by adding solutions with increasing concentrations of acetonitrile (5, 8, 10, 12, 14, 16, 18, 20, 25, 60%). Each set of TMT-labelled samples was thus separated into 10 fractions, which were then concatenated pairwise into a final set of 5 fractions (F1, 5% + 16%; F2: 8% + 18% and so on). Finally, each fraction was dried and reconstituted in a solution consisting of 2% ACN and 0.1% FA.

#### LC-MS/MS analysis

The five fractions resulting from each TPP experiment were analyzed by an LC-MS/MS system consisting of an EASY1200 chromatograph coupled to an Exploris 480 mass spectrometer (Thermo Fisher Scientific). Peptide complex mixtures were separated by using an in-house-made analytical column with the following specifications: length, 20 cm; inner diameter, 75 μm; 3 μm-C18 silica particles (Dr. Maisch). The system was equipped with a nano-spray ESI source operating at positive polarity at 1.90 kV. All the LC analyses were carried out at 0.3 μL/min flow rate and peptides were eluted by using two mobile phases: A, consisting of 2% acetonitrile and 0.1% formic acid; B, consisting of 80% acetonitrile and 0.1% formic acid.

For the analyses investigating labeling efficiency and for preliminary injections, one μL was loaded into the system and analyzed with the following gradient: from 0% mobile phase B to 4% mobile phase B in 1 min, 4–12% B in 16 min, 12–36% B in 32 min, 36–100% B in 8 min; 5 min 100% B and back to the initial conditions (0% B) in 2 min for a total run time of 63 min. Following the preliminary injections, the five fractions were analyzed by using three distinct liquid chromatography methods according to different lipophilicity of the peptide mixtures (Table 1). Finally, each gradient was followed by 5 min at 100% phase B, after which the column was subsequently brought again to 0% mobile phase B in 2 min, for a total of 140 min of chromatographic run.

**Table 1:** Liquid chromatography gradient employed during LC-MS/MS analysis

Method	Fractions	Liquid chromatography gradient							
		% B	min	% B	min	% B	min	% B	min
1	F1; F2	0–4	1	4–12	40	12–36	80	36–100	13
2	F3	0–6	1	6–14	40	14–40	80	40–100	13
3	F4; F5	0–6	1	6–16	80	16–45	80	45–100	13

For all the experiments, MS data were acquired in data-dependent acquisition. A full MS scan was acquired over the m/z range of 375–1400 at a resolution of 60,000, with the automatic gain control (AGC) at 100%; then, the 12 most abundant precursor ions were isolated with a quadrupole mass filter window of 1.6 m/z, and fragmentation was carried out at a normalized collision energy of 34%. MS/MS analysis was performed at a resolution of 30,000, and turboTMTpro option was selected. Target AGC value was set to 100% and the intensity threshold to 1.0 × 10^4^; MS/MS isolation window was 1.6 m/z. Maximum injection time was set to 50 ms for full MS scans and to 120 ms for MS/MS scans. Finally, the dynamic exclusion time was set to 20.0 s. First mass was fixed at 100.0 m/z due to the necessity of detecting TMT reporter ion masses.

#### Data processing

The acquired raw files were analyzed with Proteome Discoverer software (version 2.4.0) and searched against the same *Homo sapiens* reference database used for DIA analysis (UniProt, downloaded March 18, 2022; 20,577 entries). TMT10plex reporter ions were specified in the quantification method and TMT signals were corrected for isotope impurities based on the manufacturer’s instructions. Precursor mass tolerance was set at 15 ppm, and fragment ions were searched at 0.02 Da. Peptides were searched using the fully tryptic cleavage constraint, with up to two missed cleavages. Carbamidomethylation (C, +57.02 Da) and TMT6plex (N, K, +229.163 Da) were used as fixed modifications, and oxidation of methionine (M) was set as the variable modification. The co-isolation threshold for reporter ion quantification was set to 50%. The FDR control was set to 1% at the PSM and peptide levels.

The data exported from PD 2.4 were analyzed using the TR workflow of the TPP R package (version 3.28) to fit melting curves [30]. All the figures were created using ggplot2 (v3.5.1) included in the tidyverse package (v2.0.0), consistent with the DIA analysis workflow.

### Validation experiments

#### Western blotting (WB) assay

Total proteins were extracted, quantified, and separated on SDS-PAGE, followed by transfer onto nitrocellulose membranes. Proteins were detected using the following specific polyclonal (p) antibodies (Abs): MT-CYB (55090-1-AP) (from Proteintech, Rosemont, USA), β−Actin (AC-15) (Sigma-Aldrich, Merck, Milan, Italy), and visualized using IRDye secondary Abs (LI-COR Biosciences GmbH, Bad Homburg, Germany). Images were acquired using the Odyssey FC Imaging System. Protein bands were quantified employing Image Studio™ Lite v5.2 (LI-COR Biosciences GmbH, Bad Homburg, Germany).

#### Reactive oxygen species detection

Cells were plated and treated with 50 μM lamotrigine for 24 h. After treatment, cells were collected, and the pellet was incubated with 10 μM chloromethyl-2′,7′–dichlorofluorescein diacetate (CM-H2DCFDA, Molecular Probe, Invitrogen, Thermo Fisher, Monza, Italy) in PBS at 37 °C for 40 min. Cells were then returned to GM and incubated at 37 °C for 20 min. In the presence of ROS, fluorescence was quantified using the Attune NxT Flow Cytometer.

#### Statistical analysis

Statistical analysis was performed using Student’s *t*-test and ordinary ANOVA test through the GraphPad Prism software. All data are reported as the mean ± standard deviations (sd) of at least three experiments. *p ≤ 0.05, **p ≤ 0.01 vs control.

## Results

### DIA-based proteomic profiling

**Fig. 1:**
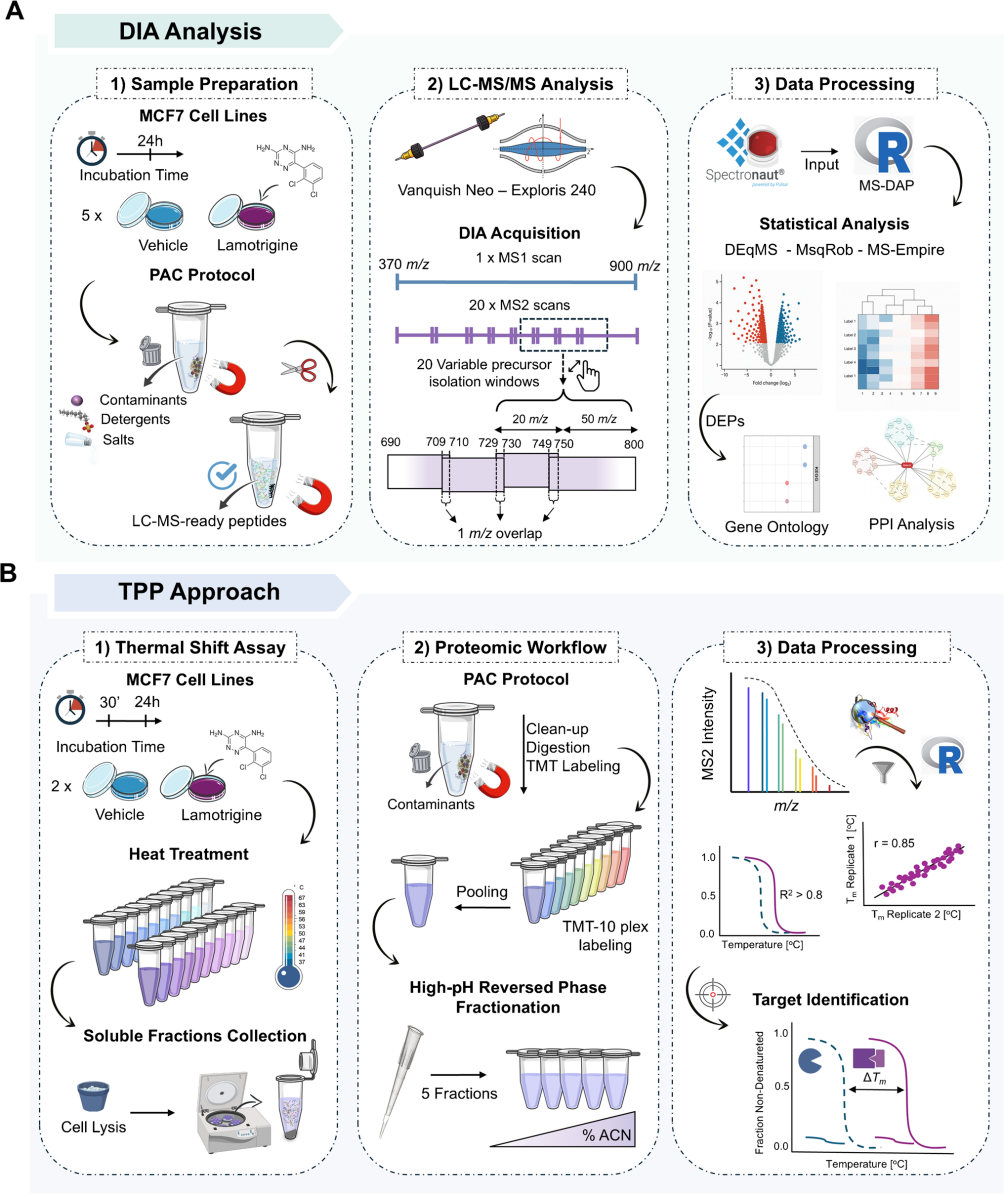
Schematic representation of the experimental workflows for proteomic analysis of lamotrigine-treated MCF-7 cells. (**A**) In the DIA-based method, cells were treated with lamotrigine or vehicle for 24 h. Proteins were extracted, digested using the PAC protocol, and analyzed by data-independent acquisition (DIA). Data were processed with Spectronaut, and differential expression assessed using the MS-DAP pipeline. Pathway enrichment analysis was conducted on the differentially expressed proteins. (**B**) In the TPP-based approach, cells were treated with lamotrigine or vehicle for 30 min and 24 h, then aliquoted and exposed to a temperature gradient (37–67 °C). Soluble protein fractions were digested using the PAC protocol, labelled with TMT 10-plex reagents, fractionated under basic-pH conditions, and analyzed by LC-MS/MS. Melting curves were fitted and thermal stability shifts (ΔTm) were calculated using the TPP R package

To investigate the pharmacological mechanisms underlying the potential antitumor effects of the well-established anticonvulsant lamotrigine, a DIA-based proteomics analysis was performed. The MCF-7 cell line, a widely used model of human breast cancer, was treated with 50 μM lamotrigine or DMSO (vehicle control) in quintuplicate. An overview of the workflow employed is presented in Fig. 1.

**Fig. 2:**
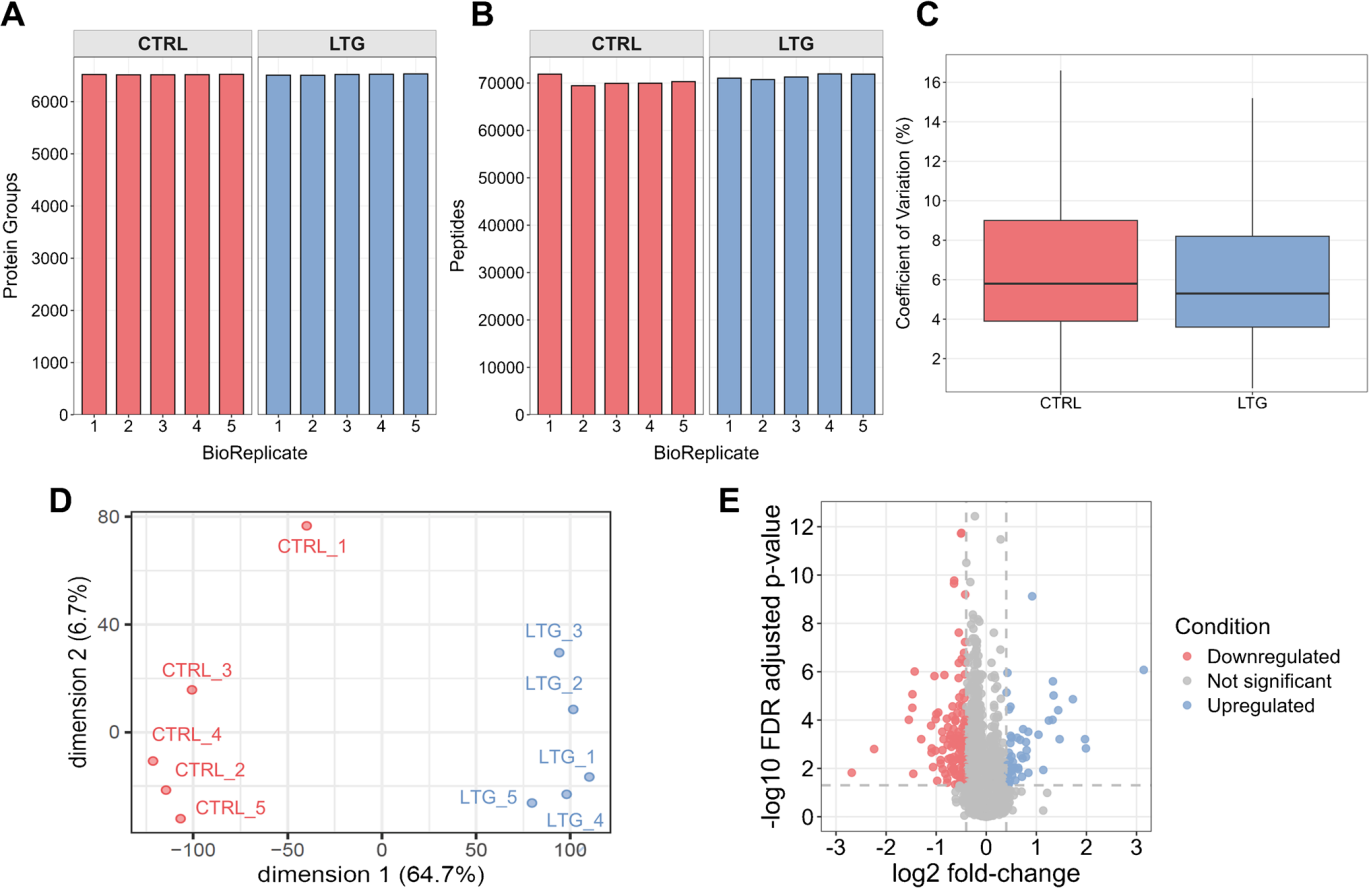
Assessment of DIA-based proteomic profiling. (**A**-**B**) Number of protein groups and peptides identified in each sample. (**C**) Coefficients of variation (CV) of protein abundances across biological replicates. (**D**) Principal component analysis (PCA) with axes representing components 1 and 2. (**E**) Volcano plot of differential protein expression. Proteins were considered significantly regulated if identified as such by at least two out of three statistical methods implemented in MS-DAP

Across all samples, a total of 75,036 peptides corresponding to 6622 proteins were identified at a 1% false discovery rate (FDR). The number of protein groups and peptides detected in each individual sample are shown in Fig. 2A and Fig. 2B, respectively. The experimental setup demonstrated high reproducibility, as reflected by the low coefficient of variation observed among biological replicates (Fig. 2C).

To comprehensively assess proteomic similarities and differences between the experimental conditions, principal component analysis (PCA) was performed. As shown in Fig. 2D, treated and control groups exhibited a clear separation along principal component 1 (PC1, 64.7% of variance explained), indicating distinct protein expression profiles.

Furthermore, a detailed analysis of differential protein expression was carried out through the MS-DAP downstream pipeline. The full output is provided in Supplementary Table S1.

To enhance statistical robustness, three complementary methods—DEqMS, MS-EmpiRe, and MsqRob—were implemented to perform pairwise comparisons across the two sample groups. The significance thresholds were set to |log₂FC|> 0.4 and adjusted *p*-value < 0.05 to identify differentially expressed proteins. The fold-change cut-off was defined based on the technical variability observed in the dataset. As estimated from the MS-DAP quality control output, the within-group standard deviation was approximately 0.2. Therefore, a cut-off corresponding to twice the standard deviation was applied to exclude proteins showing modest abundance changes. Although more stringent thresholds are often used in quantitative proteomics (e.g., fold-change > 1.5, corresponding to log₂FC ~ 0.58), lower thresholds can be justified when sufficient biological replicates are available and the data exhibit high reproducibility. In this context, a ~ 30% change in protein abundance (log₂FC ~ 0.4) can still be reliably detected, as also supported by recent DIA-based studies [32].

Using these criteria, each statistical model identified a similar number of differentially expressed proteins (DEqMS, 139; MS-EmpiRe, 146; MSqRob, 139). To further increase the robustness of the analysis, proteins were classified as differentially expressed only if identified as significant by at least two of the three methods.

**Fig. 3:**
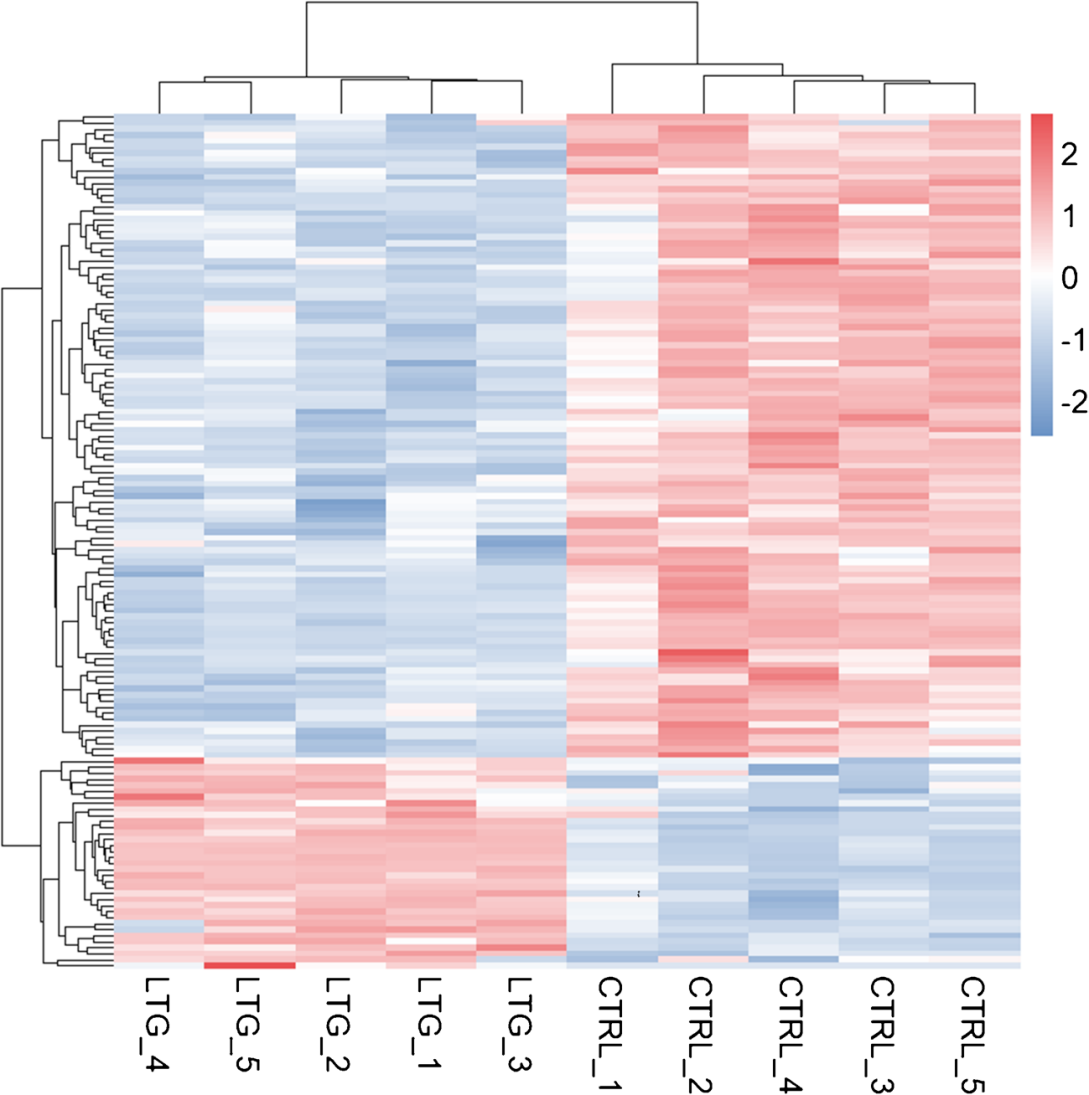
Hierarchical clustering of Z-scored protein abundances for 142 differentially expressed proteins identified after 24 h of lamotrigine treatment in the human breast cancer cell line MCF-7. Each row represents a unique protein, and each column represents a biological replicate (CTRL and LTG)

According to these requirements, a total of 142 differentially expressed proteins (DEPs) were identified after 24 h of treatment, including 107 downregulated and 35 upregulated proteins in the LTG group compared to the CTRL group (Table S2). Among these DEPs, 67 exhibited a log2FC which met more stringent criteria (> 0.58). The corresponding volcano plots illustrate their statistical significance and expression changes between conditions (Fig. 2E). Additionally, hierarchical clustering of these proteins revealed consistent differential expression patterns between LTG-treated and control samples (Fig. 3).

Among the ten most significantly up- and downregulated proteins, several were associated with biological processes essential for tumor development and maintenance, such as cell proliferation, migration, invasion, metastasis, and immune modulation. The downregulated protein set included numerous factors commonly overexpressed in breast cancer and known to support tumor progression. These encompassed cell growth and DNA replication–associated factors (DCTPP1 [33], GINS1 [34], and TRADD [35]), cytoskeletal and trafficking-associated proteins (ARMC6 [36], GIPC1 [37]), and DNA repair-associated factor (RAD23B [38]). Conversely, the upregulated group comprised proteins that have been associated with adverse clinical outcomes in cancer, including modulators of cytoskeletal remodeling and vesicular transport (RHOC [39]), chromatin organization and epigenetic regulation (MTF2 [40], JARID2 [41]), cell adhesion (FAT2 [42]), and protein synthesis (RPS27 [43]). Notably, ACAP1 [44], a GTPase-activating protein (GAP) for ARF6, which is crucial for endocytic recycling and cell surface transport, emerged as the only upregulated protein in this panel to be linked with positive clinical outcomes. In particular, elevated ACAP1 expression has been correlated with enhanced antitumor immunity and improved responses to immunotherapy [45].

To provide a comprehensive understanding of the proteomic changes associated with lamotrigine treatment, DEPs were analyzed through GO, KEGG, and Reactome pathway enrichment analyses.

**Fig. 4:**
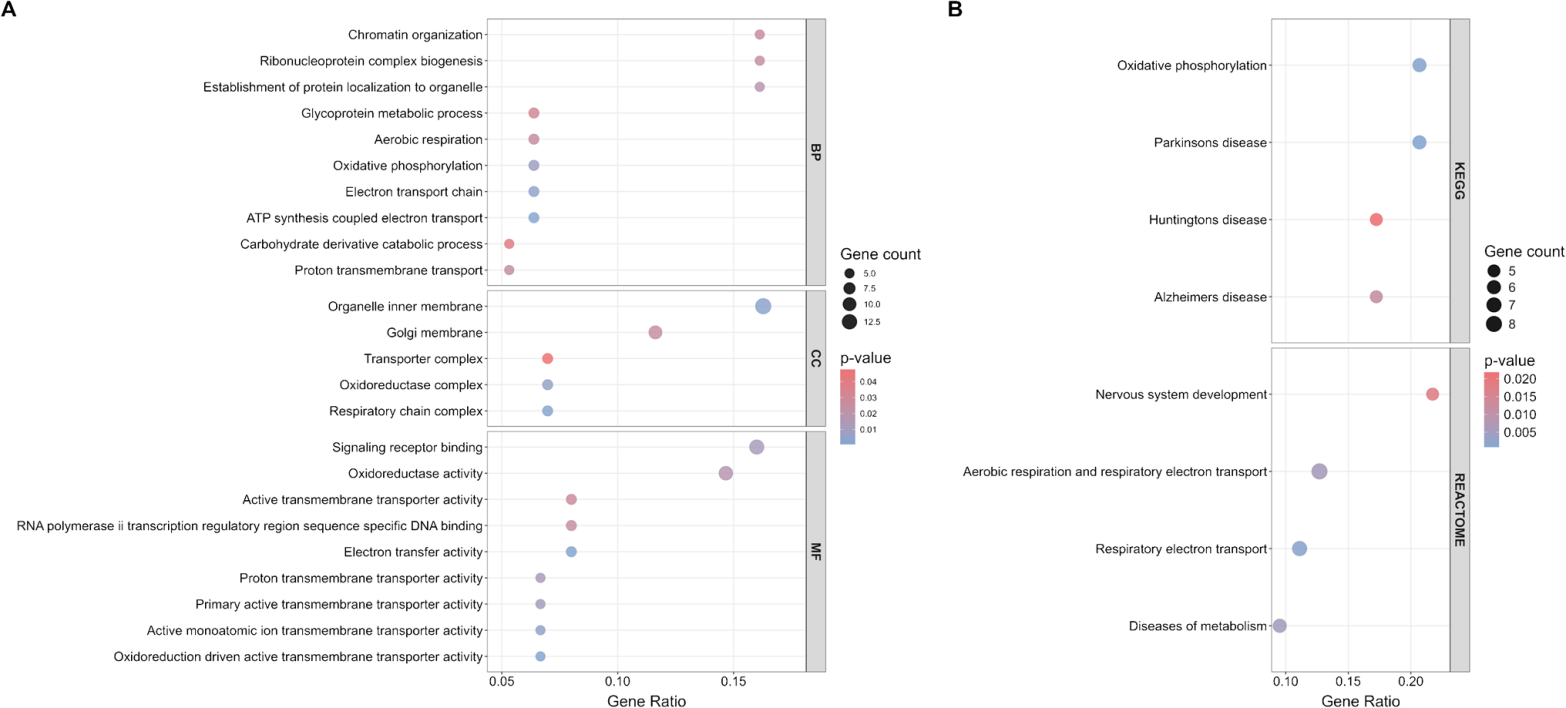
Functional enrichment analysis of differentially expressed genes. (**A**) Gene Ontology (GO) enrichment analysis displaying the top significantly enriched terms for each ontology category: biological process (BP), cellular component (CC), and molecular function (MF). (**B**) Pathway enrichment based on the KEGG and Reactome databases

GO enrichment analysis was performed across the three main ontologies: biological process, cellular component, and molecular function. Most DEPs were functionally associated with biological processes related to mitochondrial energy metabolism, including oxidative phosphorylation, electron transport chain, ATP synthesis coupled to electron transport, and aerobic respiration. Additional processes involved intracellular protein trafficking, RNA biogenesis, and chromatin remodeling. At the cellular component level, DEPs were mainly linked to mitochondrial and intracellular membrane structures, including the respiratory chain and oxidoreductase complexes, organelle inner membranes, Golgi membranes, and transporter complexes. Regarding molecular function, enriched terms were related to electron transfer, oxidoreductase activity, active transmembrane transport, and signaling receptor binding. The most significantly enriched terms from each category are shown in Fig. 4A.

KEGG enrichment analysis confirmed the involvement of oxidative phosphorylation and other mitochondrial-related pathways, some of which are annotated under neurodegenerative diseases such as Parkinson’s, Alzheimer’s, and Huntington’s disease (Fig. 4B).

Reactome pathway analysis further highlighted enrichment in respiratory electron transport, aerobic respiration, and pathways related to metabolic disorders and nervous system development, consistent with the metabolic and bioenergetic profile of the differentially expressed proteins (Fig. 4B).

**Fig. 5:**
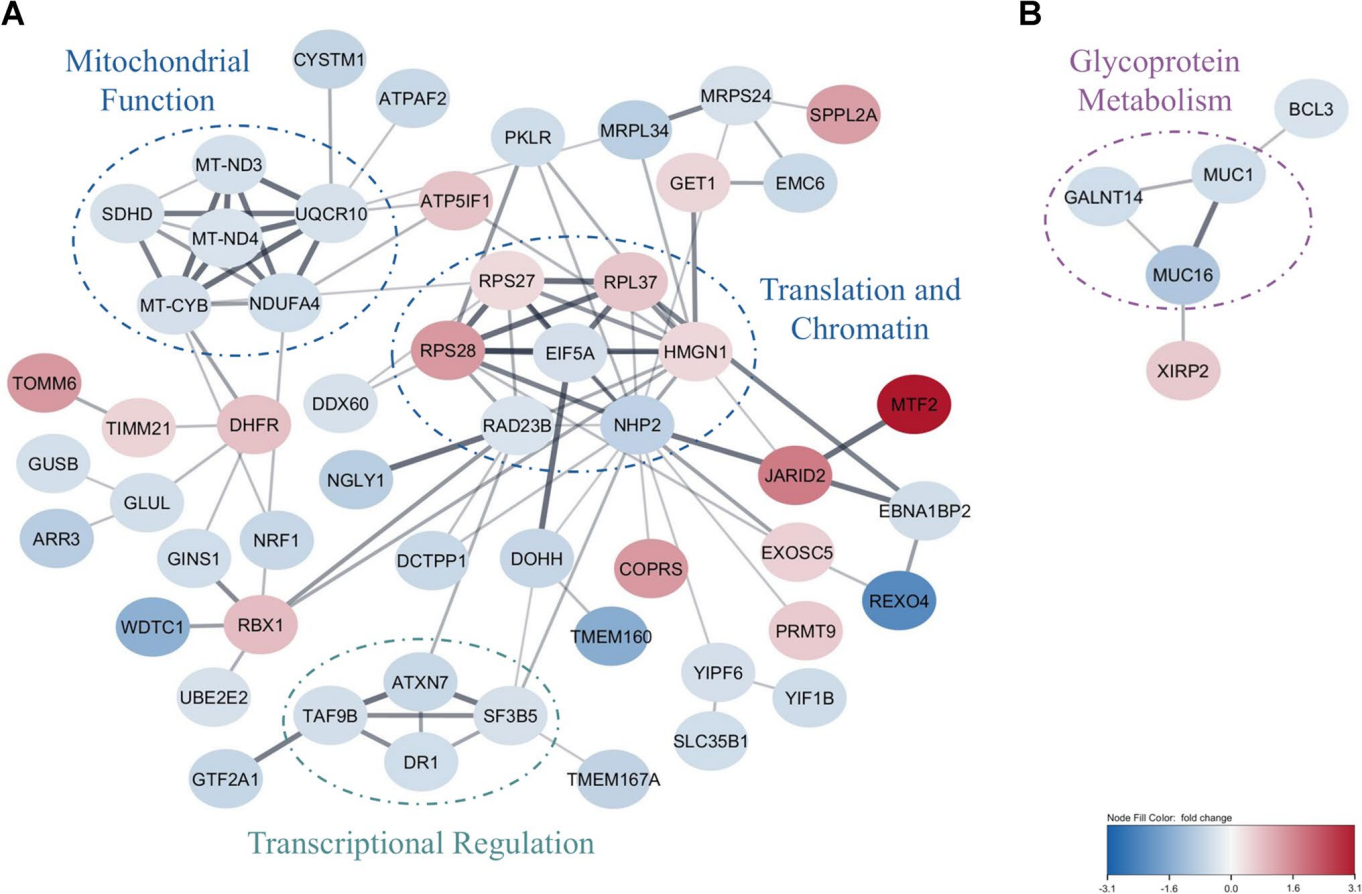
The protein-protein interaction network analysis of the DEPs. Proteins are color-coded according to their log₂ fold change (red, upregulated; blue, downregulated). (**A**) A large functional cluster highlighted (blue dashed outline) encompasses proteins involved in mitochondrial function, translation, and chromatin regulation. A second cluster (green dashed outline) contains proteins associated with transcriptional control and RNA processing. (**B**) A smaller independent cluster (purple dashed outline) includes proteins related to glycoprotein metabolism and signaling

To explore the functional associations among DEPs, a PPI network was constructed using the STRING database (confidence score ≥ 0.4) and visualized with Cytoscape. As illustrated in Fig. 5, the resulting network revealed a single major interconnected module, along with a smaller group of proteins exhibiting distinct functional relationships.

Centrality analysis highlighted several hub proteins with high degree values, including NHP2, HMGN1, RPS28, UQCR10, and RAD23B, suggesting central roles in RNA processing, chromatin regulation, ribosome function, and mitochondrial metabolism. Consistent with these findings, a prominent and functionally integrated cluster was identified, encompassing mitochondrial components, ribosomal proteins, and chromatin-associated factors, reflecting integrated regulation of mitochondrial function, protein biosynthesis, and chromatin dynamics. Within the same network, a smaller set of interacting proteins was linked to transcriptional regulation and RNA-associated chromatin activity (Fig. 5A).

In addition to the main network, an independent cluster comprising MUC1, MUC16, and GALNT14 was identified, indicating dysregulation of glycoprotein metabolism.

### Thermal proteome profiling analysis

To investigate the molecular interactions and potential targets of lamotrigine, a thermal proteome profiling (TPP) approach was employed as described by Savitski et al. [26]. In brief, MCF-7 cells were treated in duplicate with lamotrigine or vehicle for the duration of 30 minutes and 24 h, in order to monitor the changes occurring along different drug exposure time. Following the treatment, each cell culture was divided equally into 10 aliquots, which were then incubated at different temperatures, ranging from 37 to 67 °C. Cell lysis was carried out by freeze-thaw steps, and the mild detergent NP-40 was used to include membrane proteins in the investigation [30]. Insoluble protein aggregates were then removed by centrifugation, and the soluble fractions were analyzed using TMT-based multiplexed proteomics (Fig. 1B).

**Fig. 6:**
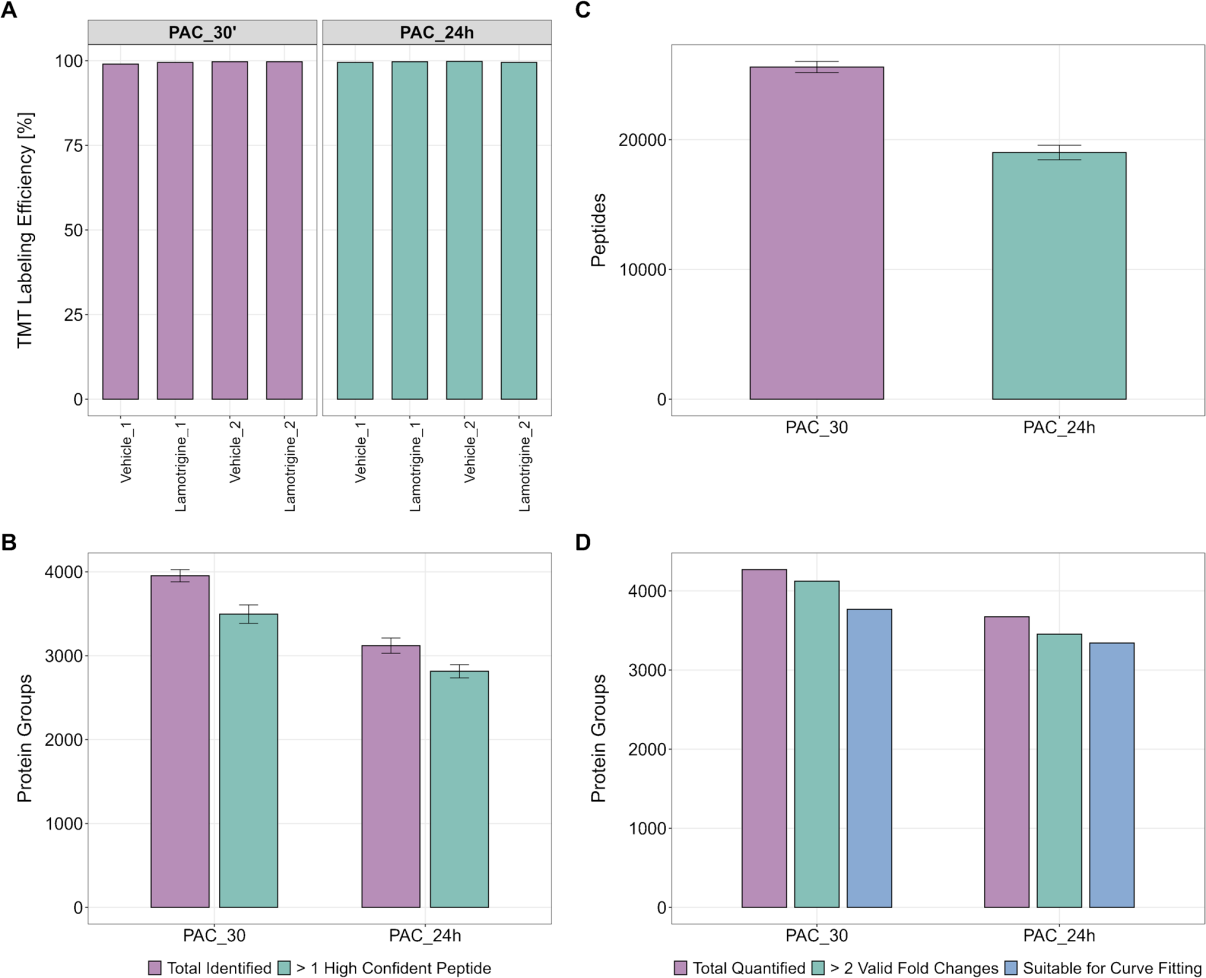
Global evaluation of TPP analysis. (**A**) Assessment of labeling efficiency in each condition. (**B**) Bar plot shows the average of protein groups identified for each experiment (pink), and the average number of quantified proteins with minimum 1 unique high-confidence peptide (blue). (**C**) The average of peptide identifications resulting for each protocol is plotted. (**D**) Bar plot shows the number of proteins suitable for statistical analysis in each protocol. The total number of quantified proteins (purple) is quality filtered for curve fitting (blue). For significance comparison, only melting curves with a *R*^2^ > 0.8 and a plateau_Vehicle < 0.3 are included (green)

Prior to quenching the TMT reaction and pooling the samples, a “label-check” step was performed to verify labeling efficiency. For both experiments, small aliquots from each single-channel TMT-labelled samples were combined and analyzed by LC-MS. Database searching was carried out by setting TMT tags on peptide N-termini and lysines as variable modifications. Since the alpha-amino group is generally the slower site to be derivatized, peptides modified with TMT reagents at peptide N-termini were considered fully labeled. Across all samples, nearly 100% efficiency was achieved (Fig. 6A).

Subsequently, the ten TMT-labelled samples were pooled into a single multiplexed peptide mixture. To reduce sample complexity prior to LC-MS/MS analysis, basic-pH reversed-phase fractionation was performed, resulting in five peptide fractions per experiment.

Protein identification and quantification were conducted using the Proteome Discoverer search engine. On average, the TPP_30’ condition yielded 3954 protein groups and 25,602 peptides, whereas TPP_24h resulted in 3121 protein groups and 19,016 peptides (Fig. 6B, C).

Subsequently, low-confidence identifications were removed, and only proteins quantified with at least one unique high-confidence peptide were included for further analysis. As a result, the number of proteins decreased by 9–13%, as shown in Fig. 6B. A detailed summary of protein group and peptide identifications for each analysis is provided in Supplementary Table S3.

Finally, the abundance values of each protein measured under 10 different temperature conditions were related to the protein quantity of the sample heated at 37 °C to calculate the relative fold changes. The acquired data were imported into R software, and the TPP-TR package was used to perform the statistical analysis (Tables S4-S5). In summary, a large portion of identified proteins, ranging from 94 to 97% resulted suitable for profiling the melting curves. Prior to performing statistical analysis, fitted sigmoidal curves were quality-filtered to ensure accurate measurement of protein melting point. In detail, two requirements were adopted: a minimum coefficient of determination (*R*^2^) greater than 0.8 and a plateau of the vehicle curve lower than 0.3 [46]. In total, TPP_30’ accurately quantified 3767 melting curves, while 3341 were obtained by TPP_24h experiment (Fig. 6D).

**Fig. 7:**
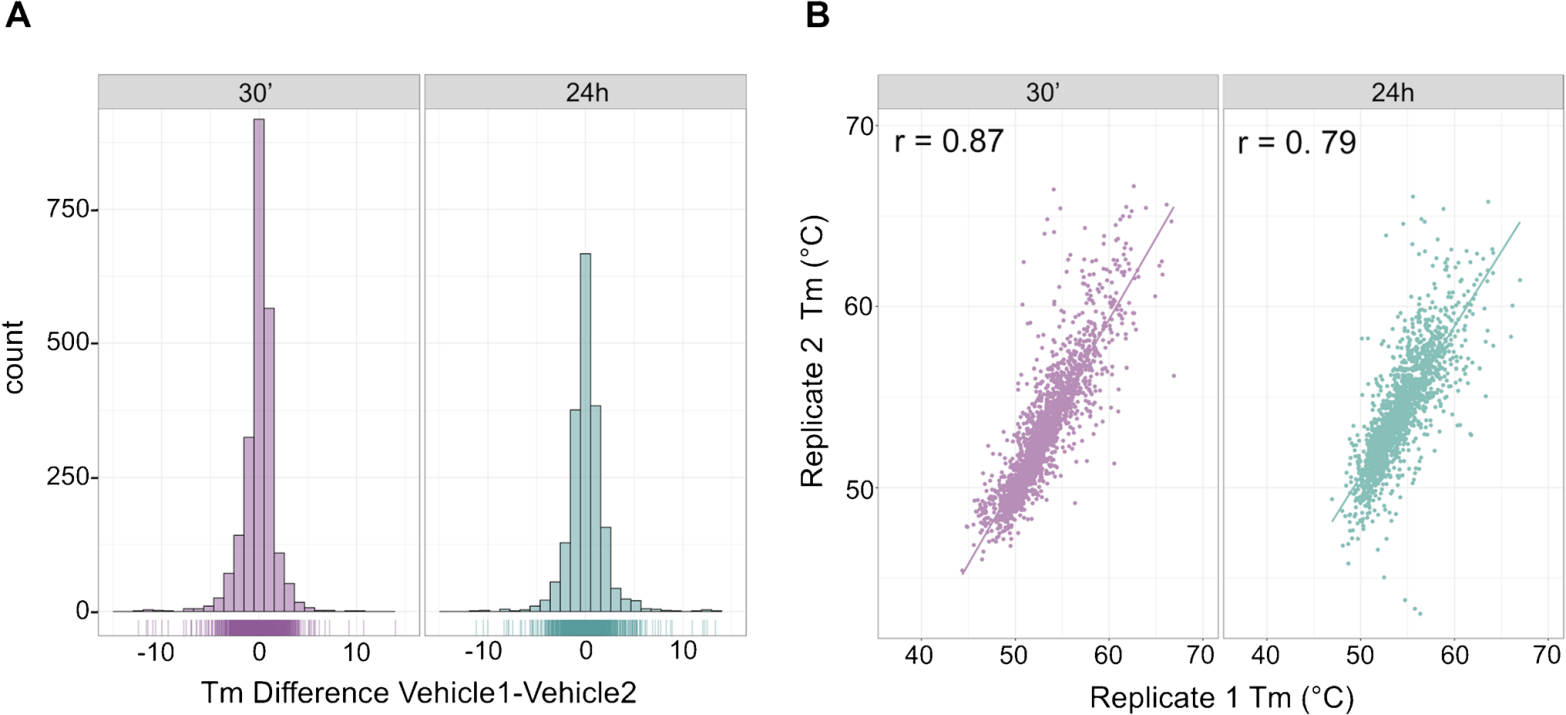
Repeatability assessment of TPP approach. (**A**) Plots show frequency distribution of measured ΔTm shifts (°C) between both vehicle experiments resulted from the 30’ (left) and 24 h (right) treatment. (**B**) Correlation of Tm of each identified protein between two biological replicates incubated with vehicle for 30’ (left) and 24 h (right) detected

The generated melting curves were then used to calculate the melting temperature of each protein. To obtain reliable results, precise and consistent Tm measurements across replicates are required. Therefore, repeatability was assessed by comparing the Tm values identified between biological replicates. As reported in Fig. 7A, both vehicle experiments demonstrated high quantitative precision, since the difference in Tm between two replicates was close to zero (TPP_30’, 0.007 ± 1.63; TPP_24h, 0.08 ± 1.85). In addition, a high correlation of 0.87 and 0.79 between the Tm values of replicates was yielded for TPP_30’ and TPP_24h, respectively (Fig. 7B). The comparison of Tm value measured in lamotrigine experiments is reported in Fig. S1.

Following the evaluation of the global performance of the TPP approach, the effect of lamotrigine on protein thermal stability was investigated.

**Fig. 8:**
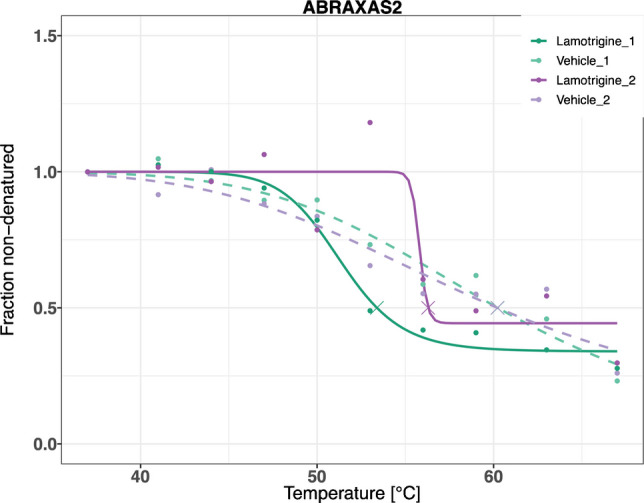
Significant change in thermal stability of ABRAXAS2 resulted by TPP_30’ experiment. The protein was destabilized in the presence of lamotrigine in both biological replicates (ΔTm_Replicate1: −6.85; ΔTm_Replicate2: −3.92)

To minimize the false positive rate, replicate experiments are required, and only proteins consistently quantified across all four datasets are considered for thermal shift significance. In total, 2655 protein groups were identified in all four sets in TPP 30’. Of these, 1536 melting curves were acquired with an *R*^2^ > 0.8 and plateau < 0.3 and thus were probed for statistical analysis. The significance of thermal shift was assessed according to the criteria established by Franken et al. [46]. Notably, only the protein ABRAXAS2 exhibited a significant change in melting temperature (Fig. 8). A consistent decrease in thermal stability was observed following lamotrigine treatment, with ΔTm shifts of − 6.85 °C and − 3.92 °C across biological replicates.

**Fig. 9:**
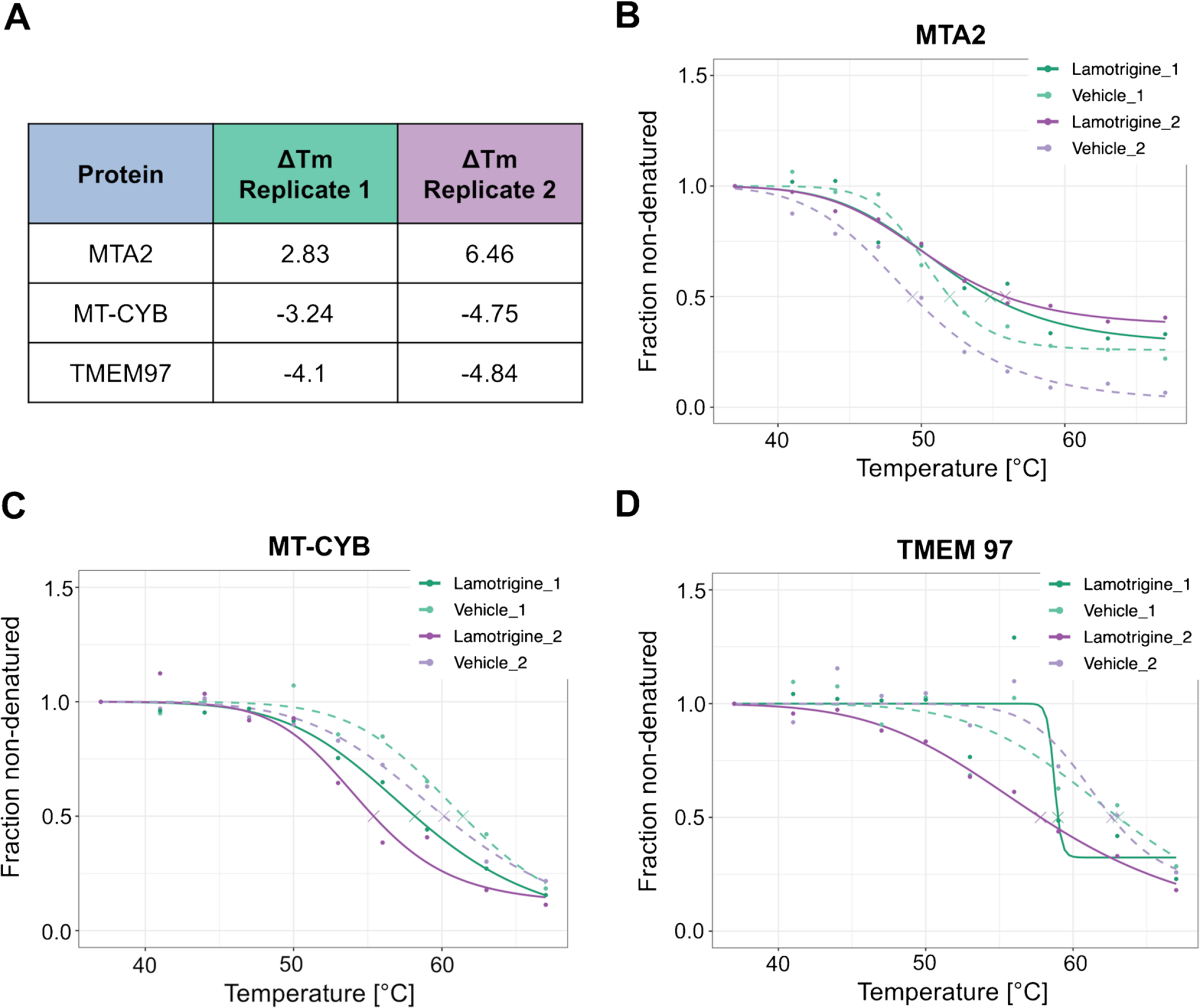
Melting curves of proteins with significant change in thermal stability resulted by TPP_24h experiment. **A** Table with significant thermal shifts of melting temperature (Tm) for MTA2 (**B**), MT-CYB (**C**), and TMEM-97 (**D**). MT-CYB and TMEM97 were destabilized in presence of the drug. Conversely, MTA2 was stabilized by the interaction with lamotrigine

For the 24 h condition, 1884 proteins were consistently quantified across all analyses, of which 1354 well-established melting curves were suitable for statistical evaluation. Using the adopted significance criteria, three proteins exhibited significant changes in melting temperature after 24 h of lamotrigine treatment (Fig. 9). In detail, MT-CYB and TMEM97 showed decreased thermal stability, whereas MTA2 displayed a positive ΔTm shift.

These proteins are involved in cancer-related processes, particularly in breast cancer. To date, none of them have been reported to physically or functionally interact with lamotrigine or other anticonvulsant drugs. The observed thermal stability shifts therefore provide new insights into potential mechanisms associated with lamotrigine exposure.

### Lamotrigine downregulates MT-CYB expression and induces oxidative stress in MCF-7 breast cancer cells

Based on the integrated DIA and TPP analyses, MT-CYB was selected for further validation, as it was significantly downregulated in the DIA dataset and exhibited decreased thermal stability in the TPP_24h condition.

**Fig. 10:**
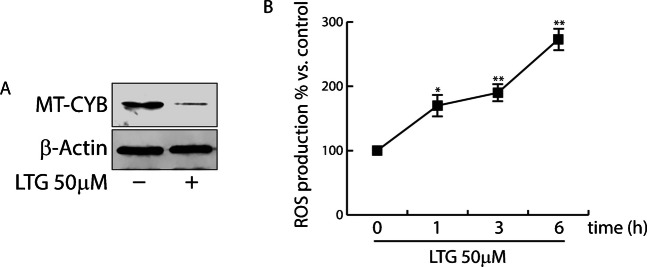
Effects of lamotrigine on MT-CYB expression and oxidative stress in MCF-7 cells. (**A**) MCF-7 cells were treated with LTG 50 µM, and MT-CYB expression was evaluated. β-Actin was used as a loading control. (**B**) MCF-7 cells treated with LTG 50 µM were assessed for ROS production. All data are reported as the mean ± standard deviations (SD) of at least three experiments. *p ≤ 0.05, **p ≤ 0.01 vs control

Protein expression levels were evaluated in MCF-7 cells after a twenty-four-hour treatment with lamotrigine 50 µM. Western blot analysis revealed a significant downregulation of MT-CYB compared to control cells, suggesting that the reduced thermal stability observed at the proteome level is accompanied by decreased protein abundance (Fig. 10A).

Given the central role of MT-CYB in the mitochondrial electron transport chain, potential functional consequences were further explored. In this context, reactive oxygen species (ROS) production was assessed as a readout of oxidative stress, revealing a marked, time-dependent increase upon lamotrigine treatment (Fig. 10B). This increase is consistent with possible involvement of mitochondrial respiratory function and progressive disruption of cellular redox homeostasis. Overall, these findings suggest alterations in mitochondrial homeostasis and support a model in which respiratory dysfunction and oxidative stress may act as upstream events contributing to lamotrigine's antitumor activity.

## Discussion

A useful strategy in the field of drug discovery is to identify novel biological targets of drugs already in clinical use, as this can significantly reduce the time and costs associated with the development of new therapies [47]. In recent years, several antiepileptic drugs have gained attention for their potential anticancer properties, particularly in breast, prostate, and other solid tumors [16]. Among these, lamotrigine, an antiepileptic agent primarily prescribed for epilepsy and bipolar disorder [15], has emerged as a promising candidate. Evidence from Morelli et al. has shown that lamotrigine exerts antitumor activity in breast cancer models, supporting the hypothesis that this drug may act beyond its established neuronal targets [17].

In order to provide a comprehensive view of lamotrigine’s action at the proteome level, two complementary mass spectrometry–based approaches were employed. DIA-based proteomics was used to assess global changes in protein abundance following drug treatment, while the TPP approach was used to investigate potential drug-protein interactions.

DIA-based proteomic analysis demonstrated that lamotrigine treatment induces significant alterations in the protein expression profile of the human breast cancer cell line MCF-7. The differentially expressed proteins are involved in key cellular processes such as energy metabolism, protein synthesis, and chromatin regulation, which are critically involved in cellular pathways commonly dysregulated in cancer, including tumor growth, survival, and treatment response.

To gain further insight into the biological implications of these proteomic changes, enrichment analyses were performed. GO, KEGG, and Reactome pathway enrichment highlighted significant perturbations in translation, glycoprotein metabolism, oxidative phosphorylation, and chromatin organization.

Consistently, PPI analysis revealed a major network enriched in mitochondrial, translational, and chromatin-associated proteins, indicating coordinated regulation of energy metabolism, protein synthesis, and epigenetic processes following lamotrigine treatment. In addition, a smaller independent cluster related to glycoprotein metabolism was identified, suggesting possible effects on post-translational modifications and signaling. 

Several ribosomal proteins involved in the translation process were significantly upregulated following lamotrigine treatment, including RPS27, RPS28, and RPS37, all components of the 40S subunit. Although ribosomal proteins are traditionally known for their structural roles in protein synthesis, growing evidence supports their extra-ribosomal functions in cancer-related processes, such as cell proliferation, apoptosis resistance, and genomic stability [48]. In particular, RPS27 and RPS28 have been implicated in tumor-promoting roles across various cancer types, supporting cell proliferation and contributing to apoptosis resistance [49, 50]. However, similar upregulation of ribosomal components has also been reported as part of cellular stress responses, including drug-induced proteotoxicity [51]. Accordingly, the increased abundance of 40S subunit proteins in lamotrigine-treated cells may represent an integrated adaptive response to proteostatic or metabolic stress, rather than a direct oncogenic effect.

Another significantly enriched pathway was oxidative phosphorylation, with several differentially downregulated proteins mapping to key mitochondrial complexes involved in electron transport and ATP production. These included NDUFA4, SDHD, UQCR10, as well as the mitochondrially encoded subunits MT-CYB, MT-ND3, and MT-ND4. Alterations in oxidative phosphorylation are a hallmark of metabolic reprogramming in cancer [52]. While many tumors exhibit a glycolytic phenotype (the Warburg effect), increasing evidence supports a role for mitochondrial respiration in sustaining tumor growth, particularly in breast cancer subtypes such as luminal and HER2-positive [51, 53]. Dysregulation of complex I (e.g., NDUFA4, MT-ND3, MT-ND4), complex II (SDHD), and complex III (UQCR10, MT-CYB) components may compromise mitochondrial function, leading to impaired ATP production, increased reactive oxygen species (ROS), and activation of cell death pathways [54, 55]. In this context, mitochondrial dysfunction may contribute to the anti-proliferative effects of lamotrigine. Supporting this, previous studies have shown that modulation of calcium signaling, a known pharmacological action of lamotrigine, can affect mitochondrial dynamics and respiratory efficiency, reinforcing the relevance of this pathway among its mechanisms of action [12].

Proteins involved in chromatin organization and epigenetic regulation were also significantly affected by lamotrigine. Notably, changes in expression were observed in factors such as MTF2 and JARID2, which are all associated with histone modification and chromatin remodeling complexes [56]. These proteins are key regulators of gene expression, cell identity, and plasticity, and have been implicated in cancer progression, stemness, and therapy resistance. The modulation of these epigenetic factors may suggest that lamotrigine exerts part of its antitumor effect through transcriptional reprogramming. This aligns with prior studies reporting that lamotrigine and other antiepileptic drugs can influence histone acetylation and chromatin accessibility, even though such effects had not yet been explored in breast cancer cells [13, 16].

Another prominent pathway enriched upon lamotrigine treatment was related to protein glycosylation, including proteins involved in N-linked and O-linked modifications. Aberrant glycosylation is a hallmark of cancer and has been implicated in multiple oncogenic processes, including immune evasion, metastasis, and resistance to therapy [57, 58]. Among the differentially expressed proteins, GALNT14, a key initiator of mucin-type O-glycosylation, was notably downregulated. This enzyme is known to modulate the glycosylation of transmembrane proteins and receptors, thereby influencing their stability, localization, and signaling capacity [59, 60]. The modulation of glycosylation-related enzymes observed in this study may suggest that lamotrigine alters the tumor cell secretory pathway or affects glycan-mediated signaling, potentially impacting cell-cell communication or immune recognition.

TPP approach interrogates the thermal stability of proteins to monitor drug-targets interactions. According to the principle of thermal shift assay, ligand binding leads to a conformational change in proteins that affects their heat resistance. Consequently, shifts in melting temperature of a protein reflect the drug engagement. Furthermore, protein thermal stability is also strongly influenced by its structural conformation and interaction network. As reported in recent studies, thermal shifts may therefore reflect not only direct drug binding, but also indirect effects on off-targets, protein complexes, or proteins whose stability is altered as part of the cellular response to treatment [61].

To explore both direct and indirect drug-protein interactions, TPP was performed at two distinct exposure times (30 min and 24 h), enabling the identification of early binding events as well as proteomic effects resulting from prolonged lamotrigine treatment. The TPP_30’ experiment revealed a single protein, ABRAXAS2, whose thermal stability significantly decreased upon lamotrigine exposure. In contrast, the TPP_24 h condition identified three proteins with altered melting temperatures, suggesting potential direct targets or proteins functionally connected to lamotrigine’s mechanism of action.

To discern whether the identified proteins represent direct lamotrigine targets or downstream effectors of cellular responses, a comparative analysis of the two TPP datasets was performed. Since a direct binder is expected to exhibit altered thermal stability across both short and long drug exposure conditions, the presence and behavior of each candidate were evaluated in both time points. Notably, ABRAXAS2 was not identified in the TPP_24h dataset, precluding further comparison. Similarly, TMEM97 was detected in the 30’ experiment, but its quantification did not allow a reliable Tm determination in all replicates. Conversely, MTA2 and MT-CYB were detected under both conditions but did not exhibit significant thermal shifts in the TPP_30’ experiment. These findings suggest that the observed alterations are more likely the result of indirect or delayed cellular responses to lamotrigine exposure, rather than direct drug-protein interactions.

It is worth noting that the limited number of significant thermal shifts observed in the TPP_30’ condition may be attributed to the short exposure time. Indeed, recent studies have reported that thirty minutes of drug incubation may be insufficient for some compounds to fully engage their intracellular targets in intact-cell TPP assays [62]. While such short durations are often effective in lysate-based experiments, where compound access to proteins is immediate, intact-cell systems introduce additional barriers such as membrane permeability, intracellular trafficking, and metabolic activation [63, 64]. Therefore, the early time point may not fully capture direct interactions, especially for targets requiring prolonged exposure to achieve measurable thermal stabilization or destabilization.

The observations resulting from the TPP analysis indicate that these proteins are affected by lamotrigine treatment, as reflected by changes in their thermal stability. However, these alterations may arise from both direct target engagement and indirect effects, including changes in protein complex formation or conformational states. By comparing the results from DIA-based proteomics and thermal proteome profiling (TPP), a notable convergence emerged at the pathway and functional module level. For instance, MT-CYB, a mitochondrially encoded subunit of respiratory complex III, was both significantly downregulated in the DIA dataset and exhibited decreased thermal stability in the TPP_24h condition, supporting its role as a potential effector of lamotrigine-induced mitochondrial stress.

In this context, evidence indicates that several antiepileptic drugs can influence mitochondrial function and cellular redox homeostasis by modulating mitochondrial membrane potential, antioxidant defense systems, and mitochondrial biogenesis [65]. These effects have been more consistently described for compounds such as valproic acid, for which an impact on mitochondrial metabolism and respiratory chain function has been demonstrated [66, 67]. In contrast, evidence regarding lamotrigine remains limited, and the specific mitochondrial protein targets underlying the observed effects have not yet been systematically identified. In this context, the present study provides an integrated mechanistic approach that identifies MT-CYB as a mitochondrial protein sensitive to lamotrigine treatment. Specifically, in MCF-7 cells, MT-CYB protein levels decrease after exposure to the antiepileptic drug. Given the central role of MT-CYB in complex III of the mitochondrial electron transport chain, these alterations suggest a potential role in impaired mitochondrial respiratory function. Consistent with this, a time-dependent increase in ROS production was observed after lamotrigine treatment, indicating a progressive disturbance of cellular redox homeostasis. Although a direct causal relationship between MT-CYB modulation and ROS accumulation cannot be established, the observed association is consistent with impaired mitochondrial function. These findings extend current knowledge of lamotrigine’s effects on mitochondrial biology, suggesting a previously unrecognized molecular axis involving MT-CYB in the cellular response to treatment. This model provides a potential mechanistic framework linking proteomic alterations, mitochondrial dysfunction, and oxidative stress, and supports further investigations aimed at elucidating the impact of lamotrigine on cellular metabolic adaptation.

Additionally, MTA2, a chromatin remodeling factor and core component of the NuRD complex, did not emerge as differentially expressed in the DIA dataset but exhibited increased thermal stability in TPP_24h. Although its expression remained unchanged, the overall enrichment of chromatin-related pathways observed in DIA analysis supports the notion that MTA2 may be functionally engaged by lamotrigine. Its thermal stabilization may reflect a structural or complex-related modulation, potentially contributing to the early epigenetic effects exerted by the drug.

This study demonstrates that lamotrigine modulates multiple cancer-associated pathways in breast cancer cells, including mitochondrial function, protein synthesis, chromatin regulation, and glycosylation. The integration of DIA and TPP analyses suggests a potential involvement of mitochondrial-related processes, with MT-CYB emerging as a key protein, in response to lamotrigine treatment. These preliminary findings provide a basis for further investigations aimed at characterizing the impact of lamotrigine on mitochondrial activity in breast cancer models.

## Supplementary Information

Below is the link to the electronic supplementary material.

Supplementary file1 (XLSX 9.75 MB)

Supplementary file2 (DOCX 163 KB)

## Declarations

### Data requirements

The mass spectrometry proteomics data have been deposited to the ProteomeXchange Consortium via the PRIDE partner repository with the dataset identifier PXD070795.
