# Supplementary material for: Integrated data-independent acquisition and thermal proteome profiling for proteomic characterization of lamotrigine-treated MCF-7 cells
Source: Anal Bioanal Chem. 2026 May 20;418(14):4497–514. doi: 10.1007/s00216-026-06540-z (PMC13375833; doi:10.1007/s00216-026-06540-z)
Supplement: Supplementary file 2 — Supplementary file2 (DOCX 163 KB) [file 216_2026_6540_MOESM2_ESM.docx]

**Analytical and Bioanalytical Chemistry**

**Research Paper**

**Supporting Information**

Integrated Data-Independent Acquisition and Thermal Proteome Profiling for Proteomic Characterization of Lamotrigine-Treated MCF-7 Cells

Annarita Giuliano1 *, Elena Ricci2, Caterina Gabriele1, Mariarosa Fava2, Sofia Spadafora2, Catia Morelli2, Diego Sisci2, Marco Gaspari1*

1Research Centre for Advanced Biochemistry and Molecular Biology, Department of Experimental and Clinical Medicine, Magna Græcia University of Catanzaro, 88100 Catanzaro, Italy

2Department of Pharmacy, Health and Nutritional Sciences, University of Calabria, 87036 Rende, Italy

*Corresponding authors: Annarita Giuliano, Email: [annarita.giuliano@studenti.unicz.it](mailto:annarita.giuliano@studenti.unicz.it); Marco Gaspari, Phone Number +39 0961 3694168, Email: [gaspari@unicz.it](mailto:gaspari@unicz.it)

**Table of Contents**

**Supplementary Figures**

**Fig. S1** Reproducibility assessment of TPP workflow for Lamotrigine Experiments.

**Supplementary Tables**

**Table S1** Output of the MS-DAP analysis, including all quantified proteins and associated statistical parameters.

**Table S2** List of significantly differentially expressed proteins (DEPs) identified by applying the defined criteria.

**Table S3.** Protein and Peptide Identification resulted by using PD 2.4.

**Table S4** Thermal proteome profiling results from TPP_30’ experiment.

**Table S5** Thermal proteome profiling results from TPP_24h experiment.


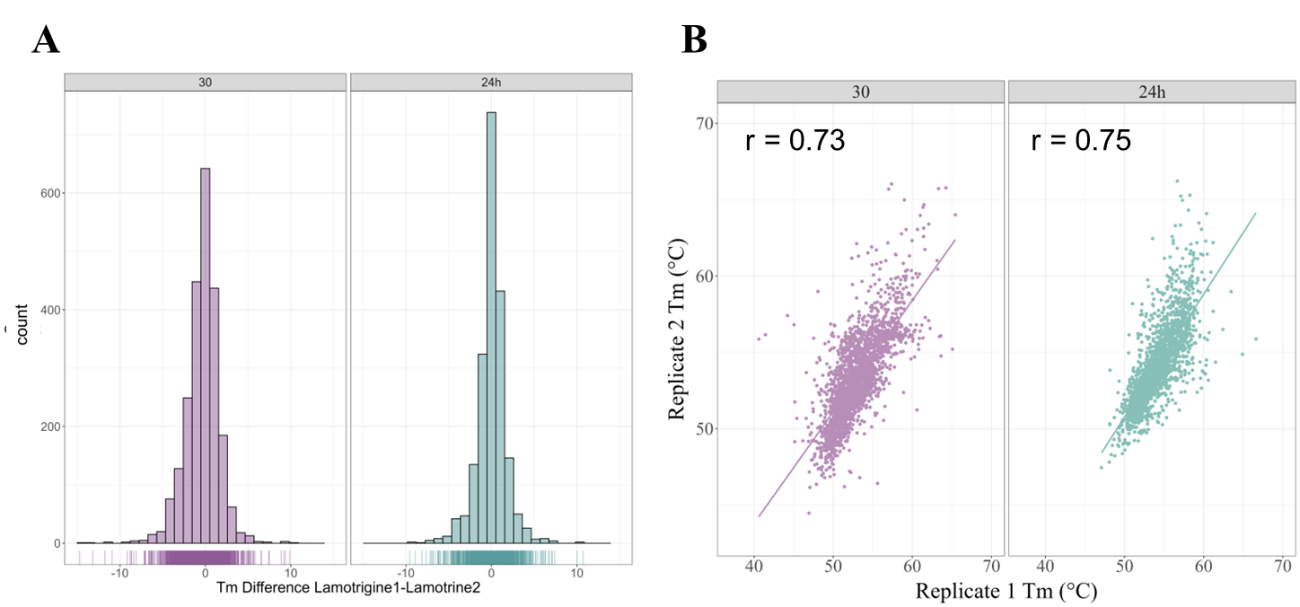


**Fig. S1** Repeatability assessment of TPP approach. (A) Plots show frequency distribution of measured ΔTm shifts (°C) between both lamotrigine experiments resulted from the 30’ (left) and 24h (right) treatment. (B) Correlation of Tm of each identified protein between two biological replicates incubated with vehicle for 30’(left) and 24h (right) detected.
